# Mental Health and Metabolic Outcomes in Early Postpartum in Women with Prediabetes After Gestational Diabetes: A Secondary Analysis of the MELINDA Trial

**DOI:** 10.3390/jcm14103592

**Published:** 2025-05-21

**Authors:** Yana Vanlaer, Caro Minschart, Karolijn Van den Keybus, Nele Myngheer, Toon Maes, Christophe De Block, Niels Bochanen, Inge Van Pottelbergh, Pascale Abrams, Wouter Vinck, Liesbeth Leuridan, Sabien Driessens, Jaak Billen, Christophe Matthys, Annick Bogaerts, Annouschka Laenen, Chantal Mathieu, Katrien Benhalima

**Affiliations:** 1 Department of Chronic Diseases and Metabolism, Clinical and Experimental Endocrinology, KU Leuven, Herestraat 49, 3000 Leuven, Belgium; yana.vanlaer@kuleuven.be (Y.V.); caro.minschart@hotmail.be (C.M.); chantal.mathieu@uzleuven.be (C.M.); 2 Faculty of Medicine, KU Leuven, Herestraat 49, 3000 Leuven, Belgium; karolijn.vandenkeybus@student.kuleuven.be; 3 Department of Endocrinology, General Hospital Groeninge Kortrijk, Campus Kennedylaan 4, 8500 Kortrijk, Belgium; nele.myngheer@azgroeninge.be; 4 Department of Obstetrics & Gynecology, Imelda Hospital, Schoolstraat 55, 2820 Bonheiden, Belgium; toon.maes@imelda.be; 5 Department of Endocrinology-Diabetology-Metabolism, Antwerp University Hospital, Wilrijkstraat 10, 2650 Edegem, Belgium; christophe.deblock@uza.be (C.D.B.); niels.bochanen@uza.be (N.B.); 6 Department of Endocrinology, AZORG Aalst, Moorselbaan 164, 9300 Aalst, Belgium; inge.van.pottelbergh@olvz-aalst.be; 7 Department of Endocrinology, ZAS Hospital Sint-Vincentius, Sint-Vincentiusstraat 20, 2018 Antwerpen, Belgium; pascale.abrams@gza.be; 8 Department of Endocrinology, ZAS Hospital Sint-Augustinus, Oosterveldlaan 24, 2610 Wilrijk, Belgium; wouter.vinck@gza.be; 9 Department of Endocrinology, General Hospital Klina, Augustijnslei 100, 2930 Brasschaat, Belgium; liesbeth.leuridan@azturnhout.be (L.L.); sabien.driessens@klina.be (S.D.); 10 Department of Laboratory Medicine, University Hospitals Leuven, Herestraat 49, 3000 Leuven, Belgium; jaak.billen@uzleuven.be; 11 Department of Chronic Diseases and Metabolism, KU Leuven, Herestraat 49, 3000 Leuven, Belgium; christophe.matthys@uzleuven.be; 12 Department of Endocrinology, University Hospitals Leuven, Herestraat 49, 3000 Leuven, Belgium; 13 REALIFE Research Group, Research Unit Woman and Child, Department of Development and Regeneration, KU Leuven, Herestraat 49, 3000 Leuven, Belgium; annick.bogaerts@kuleuven.be; 14 Faculty of Health, University of Plymouth, 3 Portland Mews, Devon PL4 8AA, UK; 15 Leuven Biostatistics and Statistical Bioinformatics Centre, KU Leuven, Herestraat 49, 3000 Leuven, Belgium; annouschka.laenen@kuleuven.be

**Keywords:** glucose intolerance, type 2 diabetes, mental health, depression, metabolic profile, gestational diabetes mellitus

## Abstract

**Aims:** To examine the association between depressive symptoms and metabolic profile in women with prior gestational diabetes mellitus (GDM) and early postpartum prediabetes, and to explore whether a mobile-based lifestyle intervention affected mental health outcomes. **Methods:** Secondary, exploratory analysis of a multi-centric randomized controlled trial (MELINDA), evaluating a mobile-based lifestyle intervention versus standard follow-up (control group) in women with prediabetes after GDM. The analysis included 166 participants who completed the Center for Epidemiologic Studies–Depression (CES-D) questionnaire [score of ≥16 being suggestive for (sub)clinical depression] at baseline (6–16 weeks postpartum) and one year post-randomization. **Results:** At one year, 26.5% of women (n = 44) reported depressive symptoms, with no significant difference between the intervention and control groups (30.5% vs. 22.6%, *p* = 0.293). Women with depressive symptoms (symptomatic women) were younger (30.9 ± 4.9 vs. 32.5 ± 3.8 years, *p* = 0.033) and were less often highly educated (61.4% vs. 80.3%, *p* = 0.028). At baseline, symptomatic women had a higher rate of metabolic syndrome (38.6% vs. 21.9%, *p* = 0.044), higher LDL-cholesterol [3.2 ± 0.8 vs. 2.8 ± 0.8 mmol/L, *p* = 0.009], lower quality of life (lower SF-36 scores, *p* < 0.050) and a higher level of anxiety based on the STAI-6 questionnaire (14.5 ± 3.6 vs. 11.2 ± 2.6, *p* < 0.001). These differences persisted at one year postpartum with worse metabolic profile, more anxiety and lower quality of life in symptomatic women. **Conclusions:** Depressive symptoms are common in women with prediabetes in early postpartum after GDM and are associated with a persistent worse metabolic profile, increased anxiety and lower quality of life postpartum. The mobile-based lifestyle intervention did not improve mental health.

## 1. Introduction

The journey into motherhood is a deep and transformative experience, marked by joy, anticipation, and a cascade of physiological changes [1,2]. For women with a recent history of gestational diabetes mellitus (GDM), the postpartum phase presents distinct challenges that go beyond the typical aspects of parenting [3,4]. GDM is not only linked to immediate health risks during pregnancy but also to long-term adverse outcomes for mothers [e.g., an increased risk of type 2 diabetes (T2DM)] and for their children (e.g., glucose intolerance, obesity and potential cognitive impairments) [4,5]. Research indicates that approximately 10% of women who give birth experience postpartum depression, with a reported range of 2.3% to 26%, and up to 50% of cases remaining undiagnosed [6]. Notably, GDM has been identified as a potential risk factor for postpartum depression, highlighting the importance of screening and suggesting a need for closer follow-up and supportive interventions for women with a history of GDM during and after pregnancy. As new mothers navigate hormonal shifts, lifestyle changes, and emotional challenges, the intricate interplay between mental health and metabolic well-being requires careful investigation [7,8].

Depressive symptoms during pregnancy are linked to increased gestational weight gain (GWG) and occur frequently in women with GDM (21.3%), often persisting postpartum with lower quality of life [9,10,11]. A few studies highlighted that physical activity during pregnancy reduces the risk of postpartum depressive symptoms, but associations between activity and depressive symptoms vary by context, highlighting the need for further research [12,13,14,15].

Women with a history of GDM and prediabetes in early postpartum are a particularly high risk group for the development of T2DM [4,5]. However, there is a lack of data on the association between depressive symptoms and the metabolic profile in early postpartum in this population. Therefore, this study aims to explore how the presence of depressive symptoms at baseline relates to metabolic health, anxiety symptoms, and quality of life one year later in women with prediabetes following a recent GDM pregnancy. This is a secondary analysis of the ‘Mobile-Based Lifestyle Intervention in Women with Glucose Intolerance after Gestational Diabetes Mellitus’ (MELINDA) trial—a large Belgian multicenter randomized controlled trial that evaluated a one-year telephone- and mobile-based lifestyle intervention. In this sub-analysis, we focus on comparisons between women with compared to women without depressive symptoms at baseline, irrespective of randomization group [16]. Additionally, we explored whether group allocation to the mobile-based lifestyle intervention had an effect on mental health outcomes at one year postpartum. To our knowledge, this is the first study to provide data on mental health including depression, anxiety and quality of life, and their relationship with the metabolic profile in women who already developed prediabetes in early postpartum.

## 2. Materials and Methods

**Study design and setting**

This is an exploratory sub-analysis of the MELINDA trial, which was previously published [16]. The MELINDA trial was a multicenter RCT designed to evaluate the efficacy and feasibility of a blended-care, telephone- and mobile-based lifestyle intervention in women with prediabetes in early postpartum after a recent pregnancy complicated by GDM. Results from the MELINDA trial indicated that while the intervention did not help participants meet weight loss goals, it reduced the risk of metabolic syndrome and sedentary behavior [16]. The study was registered at ClinicalTrials.gov as NCT03559621, approved by the Medical Ethical Committees of all participating centers (Belgian number: B322201837047), and conducted in accordance with the declaration of Helsinki. Participants provided informed consent before inclusion in the study.

GDM was diagnosed by the International Association of the Diabetes and Pregnancy Study Groups (IADPSG) criteria. Glycemic targets as recommended by the American Diabetes Association (ADA) were used for the treatment of GDM [3].

Baseline data for the MELINDA study were collected three months postpartum from a cohort of 1201 women with GDM who underwent the postpartum 75 g oral glucose tolerance test (OGTT) [16]. Women with prediabetes at the postpartum OGTT (measured between 6–12 weeks postpartum) were invited to participate in the RCT. Prediabetes was defined according to the ADA criteria: impaired fasting glucose (IFG; 5.6–6.9 mmol/L), impaired glucose tolerance (IGT; 2-h glucose value on the OGTT between 7.8–11.0 mmol/L), or both [17]. Metabolic syndrome was defined according to the International Diabetes Federation (IDF) criteria [18]. Participants were randomly assigned in a 1:1 ratio, with stratification by center and pre-pregnancy body mass index (BMI), to either the telephone- and mobile-based lifestyle intervention group or the control group [16].

The intervention arm received a comprehensive one-year lifestyle program, including one face-to-face session, monthly telephone coaching sessions, and engagement with a mobile application (MELINDA app) aimed at fostering healthy lifestyle habits, as previously described [16]. The app did not include specific features related to mental health support or guidance. Participants in the control group received general guidance on the long-term risks associated with GDM and the importance of adopting a healthy lifestyle to mitigate the risk of T2DM. They received no additional coaching but were recommended in line with normal routine, to get a yearly screening test with fasting glycemia in primary care. At the end of the study, participants received a 75 g OGTT one year post-randomization, with the same procedures as performed at the early postpartum assessment 6–16 weeks post-delivery [16].

**Study visits and measurements**

Baseline characteristics were collected through clinical examinations, self-administered questionnaires, blood samples and by extraction of medical history from the electronic medical record.

At 12 weeks postpartum, and at one year post-randomization a 75 g OGTT was conducted, involving fasting, 30, 60, and 120-min blood samples. Participants were asked to complete several self-administered questionnaires such as a self-designed questionnaire evaluating general habits and socio-economic factors [16], a Food Frequency Questionnaire (FFQ) validated for the Belgian population assessing food and beverage consumption frequency and portion sizes [19], the International Physical Activity Questionnaire (IPAQ) validated for the Belgian population quantifying physical activity across different domains [16,20], and a validated self-designed questionnaire addressing breastfeeding practices and contraception [16]. Participants’ mental health was evaluated with the Center for Epidemiologic Studies—Depression (CES-D) questionnaire. This 20-item validated questionnaire is widely used in pregnant and postpartum women to assess symptoms of (sub)clinical depression over the past 7 days. Total score on the CES-D questionnaire can range from 0 to 60, with a score of 16 or higher being suggestive for (sub)clinical depression. In addition, the Spielberger State-Trait Anxiety Inventory (STAI-6) questionnaire was used, which is a short form of the State-Trait Anxiety Inventory (STAI), a widely used psychological tool designed to measure anxiety in adults. This questionnaire includes 6 items that assess two components of anxiety. Each item is rated on a 4-point scale (e.g., “Not at all”, “Somewhat”, “Moderately”, “Very much so”), with higher scores indicating higher levels of anxiety. The validated SF-36 health survey was also used, which collects general, consistent, and user-friendly quality-of-life measures [21].

This sub-analysis aimed to compare the metabolic profile between women with symptoms of depression and women without symptoms of depression, based on the one year post-randomization CES-D questionnaire. Therefore, only women with data available from the CES-D questionnaire collected at the OGTT in early postpartum and at the 1 year post randomization OGTT were included. We evaluated data from the entire participant cohort, comparing women with and without depressive symptoms at baseline, to explore associations with mental health and metabolic outcomes at one year. In addition, we evaluated outcomes separately for the intervention and control groups, to evaluate the impact of the mobile-based lifestyle intervention on participants’ mental health and metabolic profile.

**Procedures**

The OGTTs included assessments of glucose and insulin levels at fasting, 30 min, 60 min, and 120 min. Concurrently, a fasting lipid profile (total cholesterol, triglycerides, HDL, and LDL cholesterol) and HbA1c were determined [16]. Participants were instructed to fast for at least 10 h, abstain from smoking and physical activity, and consume only water, avoiding coffee, carbonated drinks, or any beverages containing sugar or caffeine.

Glucose measurements during the OGTTs were collected locally in fluoride-containing tubes and promptly sent to the local laboratory for timely diagnosis of (pre)diabetes. Lipid profile, HbA1c, and insulin were centrally analyzed at the Leuven University Hospital (UZ Leuven) laboratory for consistency. Plasma glucose levels were determined using an automated colorimetric-enzymatic method on a Hitachi/Roche-Modular P analyzer (Basel, Switzerland). Insulin levels were measured through the immunometric ECLIA (Roche Modular E170), and HbA1c was assessed using the Tosoh Automated Glycohemoglobin Analyzer HLC-723G8 (Roche, Basel, Switzerland). Lipid levels were determined with the immunoassay analyzer Cobas 8000 (Roche, Basel, Switzerland). Coefficients of variance stood at 1% for glucose, 6% for insulin, approximately 2% for lipids, and 2% for HbA1c in the UZ Leuven Lab [16].

Various indices of insulin sensitivity, such as the Matsuda index, a well-established measure of whole-body insulin sensitivity and the homeostasis model assessment of insulin resistance (HOMA-IR), a measure of largely hepatic insulin resistance and β-cell function (HOMA-B), the insulinogenic index divided by HOMA-IR and the insulin secretion-sensitivity index-2 (ISSI-2), an OGTT-derived measure that is analogous to the disposition index obtained from the frequently sampled intravenous glucose tolerance test, were measured as previously described [16].

**Clinical examinations**

Blood pressure (BP), weight, height and waist circumference were measured as previously described [16]. Overweight was defined as a BMI ≥ 25 kg/m^2^ and obesity as a BMI ≥ 30 kg/m^2^. Early postpartum weight retention (PPWR) was defined as the difference in weight measured at the postpartum OGTT (±3 months postpartum) and the pre-pregnancy weight (self-reported weight up to one month before pregnancy or weight measured during first prenatal consultation before 12 weeks).

**Gestational weight gain**

Early weight gain was calculated as the difference in weight between the first prenatal visit and the time of the OGTT (performed between 24–28 weeks gestation). Total GWG was determined as the difference in weight from the first prenatal visit to delivery. Categories of excessive and inadequate total GWG were defined in accordance with the National Academy of Medicine (NAM) guidelines, formerly known as the Institute of Medicine (IOM) guidelines [22].

**Statistical analysis**

Given that the study was not designed or powered to detect differences in depressive symptoms between the intervention and control groups of the MELINDA study, the analyses were exploratory and primarily descriptive in nature. Frequencies and percentages were used for categorical variables, while continuous variables were expressed as means with standard deviations or medians with interquartile ranges. Group comparisons were conducted using the Mann-Whitney U test for continuous or ordinal variables and the Chi-square test or Fisher exact test for categorical variables, especially when dealing with low (<5) cell frequencies. Paired binary data were analyzed using the McNemar test.

All statistical tests were performed at a 5% two-sided significance level. Statistical analyses were conducted by statistician A. Laenen using SAS software (version 9.4 of the SAS System for Windows, 2023).

## 3. Results

**Participant flow**

**Figure 1:**
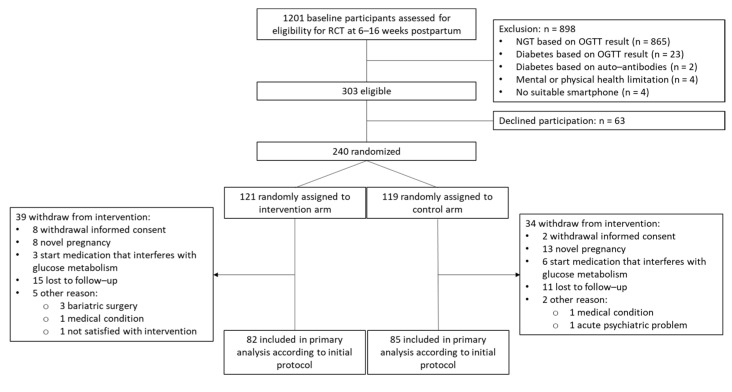
Flowchart of participants of the MELINDA study. MELINDA, Eligible participants were randomly assigned in a 1:1 ratio to either a one-year blended-care mobile-based lifestyle program (intervention) or usual care (control); RCT, randomized controlled trial; NGT, normal glucose tolerance; OGTT, oral glucose tolerance test.

Of the 240 participants randomized in the MELINDA RCT, 167 completed the study and were included in the final analysis of the MELINDA trial (82 in the intervention group and 85 in the control group). A total of 73 participants (39 in the intervention group and 34 in the control group) withdrew from the study, resulting in a dropout rate of 30.4%. The reasons for dropout have been previously described (Figure 1) [16].

**Figure 2:**
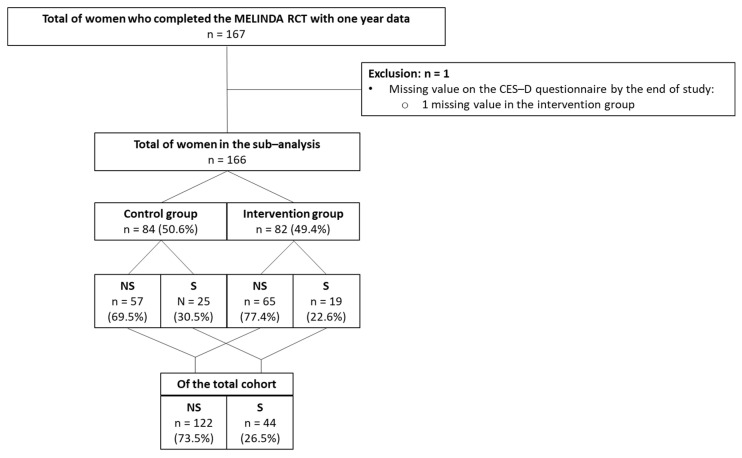
Flowchart of participants included in the secondary analysis. RCT, randomized controlled trial; CES-D questionnaire; Cener for Epidemiologic Studies-Depression questionnaire; NS, no symptoms of depression; S, symptoms of depression.

There was no difference in the prevalence of symptoms of depression at baseline between women who dropped out and women who remained in the study (*p* = 0.738). Women without data on the CES-D questionnaire were excluded (only one woman), leaving 166 women in this secondary analysis (Figure 2).

**Antidepressant use**

Among the 166 participants, 4.2% of all women (N = 7; 3 in the control group and 4 in the intervention group) were taking one or more antidepressants at baseline. Of these, four women continued using the same antidepressant medication at the one-year post-randomization visit, while three were no longer using antidepressants at that time.

**Intervention vs. control group: depressive symptoms and outcomes**

**Figure 3:**
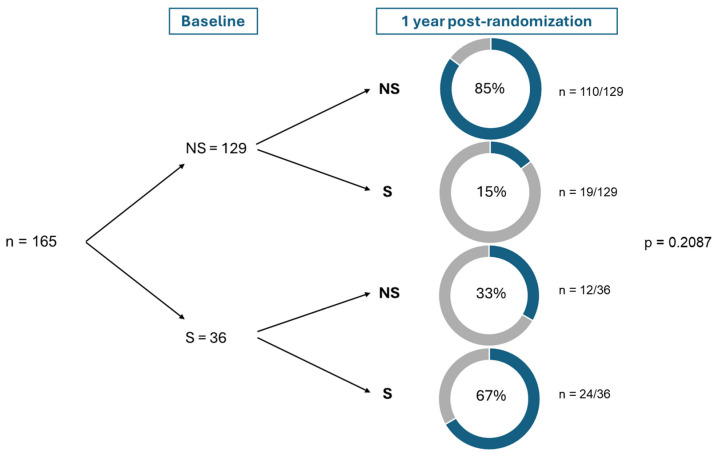
Overall change in the prevalence of depressive symptoms between baseline and one year postpartum. NS, no symptoms of depression, S symptoms of depression. The McNemar test was used for the analysis of paired binary data. Differences are considered significant at *p*-value < 0.05. One participant did not complete the CES-D questionnaire at either baseline or one year postpartum, resulting in missing data for that individual.

Of all women, 26.5% (44) women reported depressive symptoms one year post-randomization (Table 1). This was not significantly different between the intervention and control groups [30.5% (25) vs. 22.6% (19), *p* = 0.293]. There were also no significant differences between the intervention and control groups in quality of life, anxiety and metabolic outcomes such as glucose levels, insulin resistance, and lipid profile at one year postpartum (Table 2 and Table 3). Additionally, there was no significant change in the prevalence of depressive symptoms between baseline and one year postpartum (*p* = 0.2087). However, 67% of women who had depressive symptoms at baseline continued to experience them after one year, while 15% of those without depressive symptoms at baseline developed them within a year after randomization (Figure 3).

**Comparison within the total cohort**

Women with depressive symptoms were younger (30.9 ± 4.9 years vs. 32.5 ± 3.8 years, *p* = 0.033) and were less likely to have a higher education (61.4% (27) vs. 80.3% (98), *p* = 0.028) compared to women without depressive symptoms (Table 1). Women with depressive symptoms had higher fasting LDL cholesterol levels [3.2 ± 0.8 vs. 2.8 ± 0.7 mmol/L, *p* = 0.009], and a higher prevalence of a metabolic syndrome at baseline compared to women without depressive symptoms [38.6% (17) vs. 21.9% (26), *p* = 0.044]. This persisted at one year postpartum, with worse metabolic profile in women with depressive symptoms compared to women without depressive symptoms (Table 1).

In addition, both at baseline and at one year post-randomization women with depressive symptoms reported lower scores on the quality of life questionnaire (lower SF-36 scores, *p* < 0.050) and higher anxiety levels as measured by the STAI-6 questionnaire (resp. 14.5 ± 3.6 vs. 11.2 ± 2.6, *p* < 0.001 and 16.2 ± 3.6 vs. 11.2 ± 2.6, *p* < 0.001) (Table 1).

At one year post-randomization women with depressive symptoms consumed significantly less fruit (104.2 ± 89.2 vs. 136.7 ± 92.5, *p* = 0.019), compared to women without depressive symptoms. There were no significant differences in the dietary health index or other food components. No significant differences were found in physical activity between the groups with and without depressive symptoms (Table 1).

**Comparison within the intervention and control groups**

In the intervention group, women with depressive symptoms were younger (29.7 ± 4.9 years vs. 32.1 ± 3.6 years, *p* = 0.018) and were more likely to smoke (12.0% (3) vs. 0.0% (0), *p* = 0.026) than those without depressive symptoms. Moreover, they had a higher prevalence of excessive gestational weight gain (41.7% (10) vs. 17.3% (9), *p* = 0.044). At baseline, LDL cholesterol levels were significantly higher in women with depressive symptoms (3.2 ± 0.8 vs. 2.7 ± 0.7 mmol/L, *p* = 0.026). Women with depressive symptoms were also more likely to have overweight (56.0% (14) vs. 22.8% (13), *p* = 0.011) and postpartum weight retention (28.0% (7) vs. 7.0% (4), *p* = 0.029) compared to women without depressive symptoms. Furthermore, women with depressive symptoms reported lower quality of life (SF-36) scores (e.g., mental health: 67.0 ± 14.4 vs. 79.0 ± 14.9, *p* < 0.001) and higher levels of anxiety (STAI-6) scores (14.3 ± 3.0 vs. 11.2 ± 2.9, *p* < 0.001), which remained significantly different one year later (SF-36 scores: *p* < 0.050; STAI-6 scores: 16.1 ± 3.4 vs. 10.8 ± 2.5, *p* < 0.001). The prevalence of metabolic syndrome one year post-randomization was also significantly higher in women with depressive symptoms [44.0% (11) vs. 19.3% (11) compared to women without symptoms of depression, *p* = 0.030] (Table 2).

In the control group, women with depressive symptoms were less likely to have a higher education (57.9% (11) vs. 83.1% (56), *p* = 0.047) and had more often obesity compared to women without depressive symptoms (47.4% (9) vs. 32.3% (21), *p* = 0.019). The prevalence of metabolic syndrome at baseline was significantly higher in the group of women with depressive symptoms [47.4% (9) vs. 22.6% (14), *p* = 0.046], however this difference did not persist at one year post-randomization. There were no differences in other measures of metabolic profile between women with symptoms of depression and women without symptoms of depression (Table 3). Women with symptoms of depression, reported lower quality of life (SF-36) scales, and had higher levels of anxiety (STAI-6) at both baseline and one year post-randomization (resp. 14.9 ± 4.3 vs. 11.2 ± 2.3, *p* < 0.001 and 16.4 ± 4.0 vs. 11.6 ± 2.7, *p* < 0.001) compared to women without symptoms of depression (Table 3).

## 4. Discussion

Depressive symptoms occur frequently in women with GDM [11]. Women with GDM who exhibit depressive symptoms are more likely to remain depressed postpartum with lower quality of life [9,10,11]. This secondary analysis of the MELINDA trial addresses a significant knowledge gap, since limited data are available on the association between depressive symptoms and metabolic profile in early postpartum among women with prediabetes following a recent pregnancy complicated by GDM. In our study, we compared women with and without depressive symptoms at baseline, irrespective of group allocation, to evaluate their mental health and metabolic outcomes one year later.

In our study, 26.5% of women with prediabetes in early postpartum reported symptoms of depression at one year postpartum. Postpartum depression affects approximately 10–15% of women in the general population [23,24], though the prevalence can vary depending on factors such as study design and demographic characteristics. In comparison, our study found a notably higher prevalence of depressive symptoms (26.5%) in women with prediabetes following GDM, emphasizing the increased mental health burden in this high-risk group. A meta-analysis involving more than two million women showed that GDM increases the risk of postpartum depressive symptoms by 32% [25]. Women with prediabetes in early postpartum represent a particularly high risk population for developing T2DM on the long term [4,5]. To our knowledge, this is one of the first studies to focus specifically on women with prediabetes following GDM. In a systematic review by Wilson et al. (2020), the association between GDM and postpartum depression was also confirmed [26]. However, most included studies did not differentiate between glucose tolerance statuses postpartum, such as normoglycemia versus prediabetes [26]. Therefore, while their findings support an increased risk of depressive symptoms in women with a history of GDM, our results extend this knowledge by specifically identifying a higher prevalence in the subgroup with postpartum prediabetes. Our results indicate that in this high risk population, women with symptoms of depression have a persistent worse metabolic profile postpartum and have in addition also higher levels of anxiety and lower quality of life. This underscores the importance of monitoring and addressing mental health in women with prediabetes postpartum, as they may be at heightened risk for both mental health issues and long-term metabolic complications. It is therefore important that follow-up and management strategies also include strategies to improve mental health postpartum. Women with symptoms of depression are less likely to engage in a healthy lifestyle and are also less likely to adhere to a follow-up strategy to timely screen for T2DM [27,28]. The blended mobile-based lifestyle intervention as performed in the MELINDA trial, did not improve symptoms of depression. This is probably because the intervention focused mostly on promoting weight loss and tried to stimulate a healthy lifestyle with diet and increased physical activity but did not contain a targeted approach to also improve mental health. Previous studies have indicated that postpartum lifestyle interventions, particularly those not specifically targeting mental health, might have limited effects on postpartum depression [24,29,30]. The lack of significant differences between the intervention and control groups regarding depressive symptoms, quality of life, and most metabolic outcomes suggests that the intervention needs to be modified to more effectively address the psychosocial and metabolic needs of postpartum women. It may be beneficial for future interventions to integrate both mental health and physical health components, considering the bidirectional relationship between depression and metabolic risk factors. Tailoring interventions to individual needs, including demographic factors, mental health history, metabolic status, and pre-pregnancy BMI, may improve their effectiveness [31].

Studies have found that factors such as age, socio-economic status, and previous mental health history can influence the prevalence of postpartum depression [6,32,33,34,35]. We show that women with depressive symptoms were younger and less likely to have a higher education compared to women without depressive symptoms. These demographic factors have been consistently associated with a higher risk of postpartum depression in other studies, suggesting that a younger age and lower education may contribute to increased vulnerability to depressive symptoms in this population [36,37,38]. This underscores the need for interventions such as educational programs, accessible care, and community-based support. These should not only focus on mental health but also take into account socio-demographic factors that may exacerbate the risk of depression and other health issues [29,39,40].

Women with depressive symptoms had more often an adverse metabolic profile with a higher prevalence of metabolic syndrome and higher LDL cholesterol levels at baseline and this adverse metabolic profile persisted at one year post-randomization. Previous research has suggested a bidirectional relationship between depression and metabolic syndrome, where each condition potentially exacerbates the other [41,42]. This association may be mediated by factors such as inflammation, stress response, and lifestyle behaviors, which are common to both conditions [43,44]. Elevated levels of pro-inflammatory cytokines, altered cortisol responses to stress, and unhealthy lifestyle behaviors (e.g., poor diet, lack of physical activity) can contribute to both depressive symptoms and metabolic dysfunction [45,46]. In addition to these pathways, emerging evidence suggests that altered biological mechanisms—such as reduced melatonin production and decreased levels of gut-derived short-chain fatty acids like butyrate—may also play a role in both depressive symptoms and metabolic dysregulation [47,48,49,50]. These mechanisms might be particularly relevant in the postpartum period and warrant further investigation in future studies.

Women with depressive symptoms reported lower quality of life and higher anxiety levels at baseline, which persisted one year postpartum. This is in line with existing evidence that depression and anxiety often co-occur and negatively impact overall well-being and quality of life. The presence of both depressive and anxiety symptoms can lead to impaired daily functioning and reduced social engagement, which can further affect the mother’s physical and mental health [51,52].

Our findings indicate that, one year post-randomization, women with depressive symptoms consumed significantly less fruit, compared to those without depressive symptoms. This may suggest that depressive symptoms might hinder the adoption of healthier dietary habits, which could potentially worsen metabolic health. This underscores the difficulty of maintaining a balance between mental health and healthy eating habits, emphasizing the need for interventions that take the impact of mental health on eating behavior into account. Such interventions should focus on supporting women with depressive symptoms in making better health-related choices [53]. Moreover, research indicates that plant-based diets, rich in vegetables, fruits, and whole grains, improve insulin sensitivity, reduce inflammation, and reduce depressive symptoms [54]. This underscores the significance of a balanced diet for both mental and physical well-being. Similarly, the Mediterranean diet enhances mental health and cognitive function. In contrast, diets high in processed foods and sugars, which increase insulin resistance, are linked to poorer mental health, including depression and anxiety [55,56]. Still, it remains important to emphasize that mental health is the result of a complex interplay of various factors, including physical activity, social participation, sleep quality, and even financial stability [57,58].

A key strength of our study is that to our knowledge, this is the first study to examine mental health outcomes, including depression, anxiety, and quality of life, in women with prediabetes in early postpartum following a recent GDM-complicated pregnancy. Additionally, we provide comprehensive data on the association between depressive symptoms and metabolic profile in this high-risk population. However, our study has also some limitations. Our follow-up period was limited to one year, which may have been too short to observe long-term effects on mental health outcomes. Furthermore, as this is a secondary analyses the sample size may not have been adequately powered to detect significant differences between the intervention and control groups in terms of mental health outcomes. Another limitation of this study is that we could not account for participants’ history (during pregnancy) of depression. Additionally, we had no data on sleep quality. Sleep disturbances, which are common in the postpartum period, are closely linked to mental health outcomes such as depressive symptoms and quality of life and could confound the observed associations [59,60]. Future research should include measures of sleep quality to provide a more nuanced understanding of its role in the relationship between mental health and metabolic health in this high-risk population. Antidepressant use was reported by only a small subset of participants (4.2%), and more than half of them were no longer using the medication at one year. This suggests a limited impact on our findings; however, the potential influence of pharmacological treatment on mental health and metabolic outcomes cannot be entirely excluded. Finally, the MELINDA intervention was not designed to specifically address or assess mental health, which might explain why no significant improvements in depressive symptoms were observed between the intervention and control groups. Future interventions may benefit from integrating mental health support into lifestyle programs, particularly through digital platforms like mobile apps. Recent studies indicate that digital interventions can improve both mental health and lifestyle outcomes in the postpartum period. Addressing challenges like time constraints, caregiving, and disrupted sleep may enhance their effectiveness [61,62].

## 5. Conclusions

In conclusion, we show that symptoms of depression are common in women with prediabetes in early postpartum after a recent history of GDM and are associated with a persistent worse metabolic profile, more anxiety and a lower quality of life postpartum. The mobile-based lifestyle intervention did not improve mental health postpartum. This highlights the need for targeted, multifaceted approaches that address both the psychological and physical health of women with a recent history of GDM.

## Figures and Tables

**Table 1:** Comparison of women of the total cohort who had no symptoms of depression and women with symptoms of depression.

	Group with no Symptoms of Depression (<16 on CES-D Questionnaire) N = 122 (73.5%)	Group with Symptoms of Depression (≥16 on CES-D Questionnaire) N = 44 (26.5%)	*p*-Value
**General characteristics**			
Age (years) baseline	32.5 ± 3.78	30.9 ± 4.93	**0.033**
% Non-Caucasian	16.39 (20)	18.18 (8)	0.816
Highest education % None/primary school % Until age of 15 years % High school % Higher education (bachelor/master)	0.00 (0) 5.74 (7) 13.93 (17) 80.33 (98)	2.27 (1) 11.36 (5) 25.00 (11) 61.36 (27)	**0.028**
% Paid professional activity	88.52 (108)	79.55 (35)	0.201
Monthly net income family %Low income < €1500 %€1500–5000 % >€5000	2.46 (3) 86.89 (106) 10.66 (13)	6.98 (3) 88.37 (38) 4.65 (2)	0.227
% Living without partner	15.57 (19)	22.73 (10)	0.354
% Currently smoking	3.28 (4)	11.36 (5)	0.056
% Multiparity	54.10 (66)	47.73(21)	0.487
% History of GDM in previous pregnancy	21.25 (17)	23.08 (6)	1.000
% History of PCOS	5.98 (7)	2.44 (1)	0.681
% History of miscarriage	36.07 (44)	29.55 (13)	0.465
Pre-pregnancy BMI (kg/m^2^)	26.7 ± 4.93	28.0 ± 6.66	0.389
% First degree family history of T2DM	28.45 (33)	33.33 (14)	0.560
% Insulin use in pregnancy	31.15 (38)	36.36 (16)	0.575
**Gestational weight gain**			
Total Weight gain (first visit till delivery) (Kg)	8.3 ± 5.54	8.1 ± 6.25	0.808
% Excessive weight gain	17.12 (19)	28.57 (12)	0.122
% Inadequate weight gain	50.45 (56)	52.38 (22)	0.858
**Baseline (6–16 weeks postpartum)**			
% Breastfeeding	78.69 (96)	74.42 (32)	0.671
Timing OGTT (months)	2.9 ± 0.60	2.8 ± 0.76	0.185
FPG (mmol/L) 30 min glucose OGTT (mmol/L) 1-h glucose OGTT (mmol/L) 2-h glucose OGTT (mmol/L)	5.2 ± 0.57 9.0 ± 1.31 9.5 ± 1.97 8.2 ± 1.33	5.3 ± 0.62 9.0 ± 1.42 9.4 ± 2.00 7.9 ± 1.65	0.185 0.733 0.878 0.475
% Metabolic syndrome	21.85 (26)	38.64 (17)	**0.044**
BMI (kg/m^2^)	26.9 ± 4.98	28.5 ± 6.81	0.285
% Overweight (BMI 25.0–29.9) % Obese (BMI ≥ 30)	30.33 (37) 28.69 (35)	34.09 (15) 31.82 (14)	0.719
Mean systolic blood pressure (mmHg)	117.6 ± 11.85	119.8 ± 12.33	0.137
Mean diastolic blood pressure (mmHg)	74.5 ± 9.12	76.8 ± 9.14	0.204
% Hypertension (systolic BP ≥ 140 and/or diastolic BP ≥ 90 mmHg)	5.74 (7)	15.91 (7)	0.055
% Waist circumference >80 cm	80.67 (96)	79.55 (35)	1.000
% Waist circumference > 88 cm	51.26 (61)	65.91 (29)	0.112
% PPWR > 0 kg % PPWR > 5 kg	54.10 (66) 10.66 (13)	56.82 (25) 22.73 (10)	0.860 0.072
HbA1c (%)	5.4 ± 0.31	5.4 ± 0.37	0.736
HbA1c (mmol/mol)	35.6 ± 3.41	35.4 ± 4.03	0.736
Fasting total cholesterol (mmol/L) Fasting HDL-cholesterol (mmol/L) Fasting LDL-cholesterol (mmol/L) Triglycerides (mmol/L)	4.8 ± 0.81 1.6 ± 0.36 2.8 ± 0.76 1.1 ± 0.69	5.0 ± 1.06 1.5 ± 0.56 3.2 ± 0.82 1.2 ± 0.72	0.071 0.305 **0.009** 0.267
Matsuda insulin sensitivity	3.8 ± 2.12	3.8 ± 2.58	0.385
HOMA-IR	2.4 ± 1.48	3.0 ± 2.42	0.381
HOMA-B	120.9 ± 66.70	127.3 ± 66.78	0.674
ISSI-2	1.4 ± 0.39	1.5 ± 0.45	0.935
Insulinogenic index/HOMA-IR	0.2 ± 0.16	0.2 ± 0.14	0.730
FFQ Total fruit (g) Total vegetables (g) Total meat (g) Total fish (g) Total discretionary foods (g) Daily protein intake (g) Daily fat intake (g) Daily carbohydrate intake (g) Daily fiber intake (g) Daily water intake (ml) Dietary Health Index (%)	133.9 ± 104.08 204.5 ± 100.61 116.7 ± 59.45 24.3 ± 31.69 326.9 ± 290.20 66.1 ± 21.76 55.0 ± 18.47 188.8 ± 57.44 18.4 ± 6.19 2132.5 ± 531.46 75.9 ± 11.04	120.3 ± 92.63 184.1 ± 102.48 116.4 ± 56.14 20.9 ± 19.40 274.9 ± 234.04 62.2 ± 17.86 54.1 ± 17.74 174.1 ± 51.86 17.0 ± 5.58 2000.1 ± 512.84 75.5 ± 10.37	0.488 0.232 0.856 0.741 0.424 0.659 0.936 0.170 0.289 0.141 0.782
IPAQ/METs category at time of OGTT % Low % Moderate % High	11.97 (14) 43.59 (51) 44.44 (52)	17.07 (7) 39.02 (16) 43.90 (18)	0.656
SF36 Physical functioning Role physical Role emotional Emotional Wellbeing Social functioning Pain General health Vitality	88.8 ± 15.87 80.8 ± 23.52 87.6 ± 17.48 78.9 ± 12.54 90.1 ±14.98 83.0 ± 20.92 74.5 ± 15.58 62.9 ± 15.53	84.2 ± 20.33 69.6 ± 31.53 66.7 ± 27.24 65.6 ± 16.09 71.4 ± 27.92 74.2 ± 24.22 61.1 ± 19.89 52.08 ± 19.60	0.097 **0.049** **<0.001** **<0.001** **<0.001** **0.027** **<0.001** **0.002**
STAI-6	11.2 ± 2.60	14.5 ± 3.58	**<0.001**
**One year post-randomization**			
% Breastfeeding	76.23 (93)	68.18 (30)	0.319
Timing OGTT (months)	14.9 ± 0.80	14.9 ± 0.91	0.788
FPG (mmol/L) 30 min glucose OGTT (mmol/L) 1-h glucose OGTT (mmol/L) 2-h glucose OGTT (mmol/L)	5.3 ± 0.65 8.7 ± 1.49 8.8 ± 2.26 7.2 ± 2.03	5.5 ± 0.65 9.0 ± 1.75 9.2 ± 2.55 7.6 ± 2.11	0.063 0.286 0.379 0.307
% IFG % IGT % IFG + IGT	26.15 (17) 52.31 (34) 21.54 (14)	26.92 (7) 30.77 (8) 42.31 (11)	0.098
% T2DM	3.28 (4)	9.09 (4)	0.210
% Metabolic syndrome	22.31 (27)	45.45 (20)	**0.006**
BMI (kg/m^2^)	26.3 ± 5.25	27.8 ± 7.23	0.403
% Overweight (BMI 25.0–29.9) % Obese (BMI ≥ 30)	27.05 (33) 26.23 (32)	31.82 (14) 27.27 (12)	0.749
Mean systolic blood pressure (mmHg)	117.7 ± 12.65	121.9 ± 12.44	0.080
Mean diastolic blood pressure (mmHg)	76.4 ± 10.18	78.4 ± 10.52	0.212
% Hypertension (systolic BP ≥ 140 and/or diastolic BP ≥ 90 mmHg)	12.30 (15)	20.45 (9)	0.214
% Waist circumference >80 cm	62.81 (76)	72.73 (32)	0.270
% Waist circumference > 88 cm	42.98 (52)	47.73 (21)	0.600
% PPWR > 0 kg % PPWR > 5 kg	45.90 (56) 10.66 (13)	40.91 (18) 11.36 (5)	0.600
HbA1c (%)	5.3 ± 0.37	5.4 ± 0.32	0.344
HbA1c (mmol/mol)	34.7 ± 4.03	35.2 ± 3.44	0.344
Fasting total cholesterol (mmol/L) Fasting HDL-cholesterol (mmol/L) Fasting LDL-cholesterol (mmol/L) Triglycerides (mmol/L)	4.9 ± 0.97 1.5 ± 0.40 2.8 ± 0.79 1.2 ± 0.71	5.2 ± 1.55 1.4 ± 0.40 3.0 ± 0.97 1.4 ± 0.69	0.520 0.057 0.593 **0.005**
Matsuda insulin sensitivity	4.0 ± 2.21	3.5 ± 2.03	0.260
HOMA-IR	2.5 ± 1.78	3.7 ± 3.79	0.178
HOMA-B	118.8 ± 52.54	131.8 ± 75.19	0.550
ISSI-2	1.7 ± 0.69	1.5 ± 0.58	0.117
Insulinogenic index/HOMA-IR	0.2 ± 0.20	0.2 ± 0.11	0.225
FFQ Total fruit (g) Total vegetables (g) Total meat (g) Total fish (g) Total discretionary foods (g) Daily protein intake (g) Daily fat intake (g) Daily carbohydrate intake (g) Daily fiber intake (g) Daily water intake (ml) Dietary Health Index (%)	136.7 ± 92.54 209.5 ± 93.67 105.8 ± 49.92 20.3 ± 19.69 268.6 ± 242.78 58.3 ± 16.08 48.4 ± 15.82 161.1 ± 50.43 16.7 ± 5.15 2008.1 ± 555.40 77.5 ± 9.76	104.2 ± 89.21 187.4 ± 107.21 87.6 ± 53.61 22.6 ± 21.13 239.1 ± 208.66 53.4 ± 16.28 45.8 ± 18.64 152.1 ± 46.32 15.0 ± 4.94 1773.9 ± 585.64 75.4 ± 9.12	**0.019** 0.168 **0.009** 0.455 0.667 0.055 0.188 0.414 0.051 **0.014** 0.097
IPAQ/METs category at time of OGTT % Low % Moderate % High	8.26 (9) 41.28 (45) 50.46 (55)	8.11 (3) 32.43 (12) 59.46 (22)	0.620
SF36 Physical functioning Role physical Role emotional Vitality Mental health Social functioning Pain General health	91.4 ± 11.01 75.9 ± 16.70 86.4 ± 16.81 83.3 ± 12.22 74.6 ± 11.77 63.3 ± 16.99 77.2 ± 15.57 56.7 ± 13.15	83.3 ± 20.80 59.2 ± 23.64 55.3 ± 22.67 59.5 ± 15.40 50.5 ± 12.38 36.7 ± 21.12 61.6 ± 19.16 40.1 ± 14.04	**0.001** **<0.001** **<0.001** **<0.001** **<0.001** **<0.001** **<0.001** **<0.001**
STAI-6	11.2 ± 2.59	16.2 ± 3.63	**<0.001**

GDM: gestational diabetes mellitus; PCOS: polycystic ovary syndrome; BMI: body mass index; T2DM: type 2 diabetes mellitus; OGTT: oral glucose tolerance test; BP: blood pressure; IFG: impaired fasting glucose; IGT: impaired glucose tolerance; PPWR: postpartum weight retention; HDL: high density lipoprotein cholesterol; LDL-cholesterol: low density lipoprotein cholesterol; HOMA-IR: Homeostasis Model of Assessment—Insulin Resistance; HOMA-B: Homeostasis Model of Assessment—Beta-cell Function; ISSI-2: insulin secretion-sensitivity index-2; FFQ: Food Frequency Questionnaire; IPAQ: International Physical Activity Questionnaire; METs: metabolic equivalent of task [MET] minutes/week; CES-D: Center for Epidemiologic Studies–Depression; SF-36: quality-of-life questionnaire; STAI-6: Spielberger State-Trait Anxiety Inventory. Categorical variables are presented as frequencies % (n); continuous variables are presented as mean ± SD if normally distributed and as median ± IQR if not normally distributed; Differences are considered significant at *p*-value < 0.05. Bold means a statistical significant value of *p* < 0.05.

**Table 2:** Comparison of women of the intervention group who had no symptoms of depression and women with symptoms of depression.

	Group with no Symptoms of Depression (<16 on CES-D Questionnaire) N = 57 (69.5%)	Group with Symptoms of Depression (≥16 on CES-D Questionnaire) N = 25 (30.5%)	*p*-Value
**General characteristics**			
Age (years) baseline	32.1 ± 3.57	29.7 ± 4.85	**0.018**
% Non-Caucasian	14.04 (8)	12.00 (3)	1.000
Highest education % Until age of 15 years % High school % Higher education (bachelor/master)	7.02 (4) 15.79 (9) 77.19 (44)	12.00 (3) 24.00 (6) 64.00 (16)	0.431
% Paid professional activity	91.23 (52)	84.00 (21)	0.445
Monthly net income family %Low income < €1500 %€1500–5000 % >€5000	0.00 (0) 87.72 (50) 12.28 (7)	4.00 (1) 92.00 (23) 4.00 (1)	0.199
% Living without partner	14.04 (8)	24.00 (6)	0.341
% Currently smoking	0.00 (0)	12.00 (3)	**0.026**
% Multiparity	49.12 (28)	48.00 (12)	1.000
% History of GDM in previous pregnancy	27.27 (9)	7.14 (1)	0.242
% History of PCOS	9.09 (5)	0.00(0)	0.316
% History of miscarriage	26.32 (15)	24.00 (6)	1.000
Pre-pregnancy BMI (kg/m^2^)	26.0 ± 5.19	27.4 ± 5.31	0.194
% First degree family history of T2DM	27.27 (15)	21.74 (5)	0.778
% Insulin use in pregnancy	26.32 (15)	32.00 (8)	0.604
**Gestational weight gain**			
Total Weight gain (first visit till delivery) (Kg)	8.11 ± 4.561	9.74 ± 5.730	0.241
% Excessive weight gain	17.31 (9)	41.67 (10)	**0.044**
% Inadequate weight gain	53.85 (28)	37.50 (9)	0.222
**Baseline (6–16 weeks postpartum)**			
% Breastfeeding	73.68 (42)	68.00 (17)	0.604
Timing OGTT (months)	2.8 ± 0.63	2.9 ± 0.87	0.910
FPG (mmol/L) 30 min glucose OGTT (mmol/L) 1-h glucose OGTT (mmol/L) 2-h glucose OGTT (mmol/L)	5.2 ±0.56 9.1 ±1.25 9.7 ±2.04 8.4 ±1.34	5.3 ±0.62 8.9 ±1.46 8.9 ±2.14 7.8 ±1.85	0.506 0.658 0.201 0.238
% Metabolic syndrome	21.05 (12)	32.00 (8)	0.402
BMI (kg/m^2^)	26.1 ± 5.26	28.1 ± 5.54	0.110
% Overweight (BMI 25.0–29.9) % Obese (BMI ≥ 30)	22.81 (13) 24.56 (14)	56.00 (14) 20.00 (5)	**0.011**
Mean systolic blood pressure (mmHg)	116.5 ± 11.16	118.4 ± 12.80	0.414
Mean diastolic blood pressure (mmHg)	74.5 ± 7.02	76.2 ± 9.31	0.455
% Hypertension (systolic BP ≥ 140 and/or diastolic BP ≥ 90 mmHg)	3.51 (2)	12.00 (3)	0.163
% Waist circumference >80 cm	73.68 (42)	84.00 (21)	0.400
% Waist circumference > 88 cm	40.35 (23)	64.00 (16)	0.058
% PPWR > 0 kg % PPWR > 5 kg	50.88 (29) 7.02 (4)	64.00 (16) 28.00 (7)	0.338 **0.029**
HbA1c (%)	5.4 ± 0.25	5.3 ± 0.27	0.183
HbA1c (mmol/mol)	35.8 ± 2.68	34.8 ± 2.90	0.183
Fasting total cholesterol (mmol/L) Fasting HDL-cholesterol (mmol/L) Fasting LDL-cholesterol (mmol/L) Triglycerides (mmol/L)	4.7 ± 0.75 1.6 ± 0.32 2.7 ± 0.65 1.1 ± 0.61	4.9 ± 1.14 1.6 ± 0.67 3.2 ± 0.82 1.2 ± 0.85	0.160 0.553 **0.026** 0.553
Matsuda insulin sensitivity	3.9 ± 2.14	4.1 ± 2.77	0.769
HOMA-IR	2.3 ± 1.62	2.7 ± 2.03	0.526
HOMA-B	118.0 ± 59.46	124.9 ± 60.94	0.613
ISSI-2	1.5 ± 0.45	1.6 ± 0.49	0.761
Insulinogenic index/HOMA-IR	0.3 ± 0.20	0.2 ± 0.14	0.526
FFQ Total fruit (g) Total vegetables (g) Total meat (g) Total fish (g) Total discretionary foods (g) Daily protein intake (g) Daily fat intake (g) Daily carbohydrate intake (g) Daily fiber intake (g) Daily water intake (ml) Dietary Health Index (%)	138.0 ± 100.19 218.9 ± 99.50 120.3 ± 60.01 26.7 ± 41.51 335.5 ± 309.23 66.4 ± 22.48 52.9 ± 19.45 186.3 ± 53.51 18.5 ± 5.78 2144.0 ± 510.70 76.2 ± 9.82	113.4 ± 89.06 178.9 ± 107.46 119.9 ± 55.68 24.4 ± 23.87 321.2 ± 255.40 61.3 ± 17.80 54.6 ±16.99 175.1 ± 53.81 16.9 ± 5.85 1996.5 ± 508.38 73.8 ± 9.89	0.308 0.096 0.774 0.828 0.968 0.559 0.319 0.344 0.370 0.251 0.311
IPAQ/METs category at time of OGTT % Low % Moderate % High	10.71 (6) 42.86 (24) 46.43 (26)	16.00 (4) 44.00 (11) 40.00 (10)	0.710
SF36 Physical functioning Role physical Role emotional Vitality Mental health Social functioning Pain General health	91.8 ± 7.71 84.9 ± 19.62 88.0 ± 17.96 64.6 ± 14.91 79.0 ± 14.87 91.0 ± 11.75 86.2 ± 19.33 76.8 ± 14.31	84.6 ± 20.00 71.3 ± 29.15 69.3 ± 22.91 53.3 ± 18.05 67.0 ± 14.43 74.0 ± 27.24 73.4 ± 23.24 63.3 ± 19.60	0.247 **0.040** **<0.001** **0.010** **<0.001** **0.003** **0.002** **0.010**
STAI-6	11.2 ± 2.91	14.3 ± 3.02	**<0.001**
**One year post-randomization**			
% Breastfeeding	70.18 (40)	64.00 (16)	0.613
Timing OGTT (months)	14.8 ± 0.71	14.9 ± 0.98	0.935
FPG (mmol/L) 30 min glucose OGTT (mmol/L) 1-h glucose OGTT (mmol/L) 2-h glucose OGTT (mmol/L)	5.2 ± 0.46 8.7 ± 1.34 9.1 ± 1.93 7.1 ± 1.75	5.3 ± 0.59 8.7 ± 1.86 8.9 ± 3.12 7.4 ± 2.55	0.579 0.786 0.487 0.932
% IFG % IGT % IFG + IGT	22.58 (7) 58.06 (18) 19.35 (6)	25.00 (3) 33.33 (4) 41.67 (5)	0.273
% T2DM	0.00 (0)	12.00 (3)	**0.026**
% Metabolic syndrome	19.30 (11)	44.00 (11)	**0.030**
BMI (kg/m^2^)	25.3 ± 5.43	27.0 ± 5.65	0.194
% Overweight (BMI 25.0–29.9) % Obese (BMI ≥ 30)	17.54 (10) 24.56 (14)	56.00 (14) 12.00 (3)	**0.003**
Mean systolic blood pressure (mmHg)	117.7 ± 12.52	120.4 ± 12.93	0.487
Mean diastolic blood pressure (mmHg)	77.7 ± 11.11	76.9 ± 11.69	0.661
% Hypertension (systolic BP ≥ 140 and/or diastolic BP ≥ 90 mmHg)	15.79 (9)	16.00 (4)	1.000
% Waist circumference >80 cm	49.12 (28)	80.00 (20)	**0.014**
% Waist circumference > 88 cm	35.09 (20)	44.00 (11)	0.468
% PPWR > 0 kg % PPWR > 5 kg	38.60 (22) 3.51 (2)	44.00 (11) 16.00 (4)	0.807 0.067
HbA1c (%)	5.3 ± 0. 26	5.33 ± 0.27	0.982
HbA1c (mmol/mol)	34.5 ± 2.80	34.8 ± 2.92	0.982
Fasting total cholesterol (mmol/L) Fasting HDL-cholesterol (mmol/L) Fasting LDL-cholesterol (mmol/L) Triglycerides (mmol/L)	4.8 ± 1.07 1.6 ± 0.36 2.8 ± 0.84 1.1 ± 0.46	5.1 ± 1.68 1.4 ± 0.44 2.8 ± 0.71 1.3 ± 0.67	0.827 0.123 0.945 0.186
Matsuda insulin sensitivity	4.0 ± 2.03	3.9 ± 1.97	0.978
HOMA-IR	2.3 ± 1.47	3.1 ± 2.51	0.838
HOMA-B	119.1 ± 51.99	126.3 ± 67.02	0.875
ISSI-2	1.7 ± 0.64	1.6 ± 0.65	0.658
Insulinogenic index/HOMA-IR	0.2 ± 0.14	0.2 ± 0.13	0.527
FFQ Total fruit (g) Total vegetables (g) Total meat (g) Total fish (g) Total discretionary foods (g) Daily protein intake (g) Daily fat intake (g) Daily carbohydrate intake (g) Daily fiber intake (g) Daily water intake (ml) Dietary Health Index (%)	136.3 ± 90.29 208.2 ± 88.18 108.0 ± 45.49 21.2 ± 19.27 217.6 ± 200.50 58.1 ± 14.09 46.6 ± 15.57 152.0 ± 41.16 16.4 ± 4.79 1966.7 ± 506.31 79.0 ± 7.80	102.4 ± 106.03 193.9 ± 104.35 78.6 ± 42.12 27.1 ± 25.72 251.0 ± 207.61 51.1 ± 16.22 45.0 ± 14.81 152.7 ± 4.48 15.2 ± 5.12 1849.7 ± 566.24 76.8 ± 8.06	**0.031** 0.489 **0.003** 0.444 0.426 **0.043** 0.680 0.717 0.253 0.349 0.290
IPAQ/METs category at time of OGTT % Low % Moderate % High	1.89 (1) 43.40 (23) 54.72 (29)	5.00 (1) 35.00 (7) 60.00 (12)	0.539
SF36 Physical functioning Role physical Role emotional Vitality Mental health Social functioning Pain General health	92.6 ± 7.33 78.5 ± 15.22 87.6 ± 15.60 83.3 ± 12.29 76.1 ± 11.25 64.7 ± 15.87 79.0 ± 15.26 58.4 ± 11.69	85.4 ± 16.33 56.5 ± 23.49 54.0 ± 21.93 61.0 ± 14.35 50.0 ± 12.20 36.5 ± 19.74 64.0 ± 17.32 38.2 ± 16.32	**0.003** **<0.001** **<0.001** **<0.001** **<0.001** **<0.001** **<0.001** **<0.001**
STAI-6	10.8 ± 2.47	16.1 ± 3.40	**<0.001**

GDM: gestational diabetes mellitus; PCOS: polycystic ovary syndrome; BMI: body mass index; T2DM: type 2 diabetes mellitus; OGTT: oral glucose tolerance test; BP: blood pressure; IFG: impaired fasting glucose; IGT: impaired glucose tolerance; PPWR: postpartum weight retention; HDL: high density lipoprotein cholesterol; LDL-cholesterol: low density lipoprotein cholesterol; HOMA-IR: Homeostasis Model of Assessment—Insulin Resistance; HOMA-B: Homeostasis Model of Assessment—Beta-cell Function; ISSI-2: insulin secretion-sensitivity index-2; FFQ: Food Frequency Questionnaire; IPAQ: International Physical Activity Questionnaire; METs: metabolic equivalent of task [MET] minutes/week; CES-D: Center for Epidemiologic Studies–Depression; SF-36: quality-of-life questionnaire; STAI-6: Spielberger State-Trait Anxiety Inventory. Categorical variables are presented as frequencies % (n); continuous variables are presented as mean ± SD if normally distributed and as median ± IQR if not normally distributed; Differences are considered significant at *p*-value < 0.05. Bold means a statistical significant value of *p* < 0.05.

**Table 3:** Comparison of women in the control group who had no symptoms of depression and women with symptoms of depression.

	Group with no Symptoms of Depression (<16 on CES-D Questionnaire) N = 65 (77.4%)	Group with Symptoms of Depression (≥16 on CES-D Questionnaire) N = 19 (22.6%)	*p*-Value
**General characteristics**			
Age (years) baseline	32.9 ± 3.94	32.5 ± 4.68	0.715
% Non-Caucasian	18.46 (12)	26.32 (5)	0.520
Highest education % None/primary school % Until age of 15 years % High school % Higher education (bachelor/master)	0.00 (0) 4.62 (3) 12.31 (8) 83.08 (56)	5.26 (1) 10.53 (2) 26.32 (5) 57.89 (11)	**0.047**
% Paid professional activity	86.15 (56)	73.68 (14)	0.291
Monthly net income family %Low income < €1500 %€1500–5000 % >€5000	4.62 (3) 86.15 (56) 9.23 (6)	11.11 (2) 83.33 (15) 5.56 (1)	0.534
% Living without partner	16.92 (11)	21.05 (4)	0.736
% Currently smoking	6.15 (4)	10.53 (2)	0.614
% Multiparity	58.46 (38)	47.37 (9)	0.439
% History of GDM in previous pregnancy	17.02 (8)	41.67 (5)	0.113
% History of PCOS	3.23 (2)	5.88 (1)	0.522
% History of miscarriage	44.62 (29)	36.84 (7)	0.607
Pre-pregnancy BMI (kg/m^2^)	27.2 ± 4.67	28.8 ± 8.19	0.673
% First degree family history of T2DM	29.51 (18)	47.37 (9)	0.173
% Insulin use in pregnancy	35.38 (23)	42.11 (8)	0.600
**Gestational weight gain**			
Total Weight gain (first visit till delivery) (Kg)	8.5 ± 6.31	5.9 ± 6.38	0.103
% Excessive weight gain	16.95 (10)	11.11 (2)	0.721
% Inadequate weight gain	47.46 (28)	72.22 (13)	0.104
**Baseline (6–16 weeks postpartum)**			
% Breastfeeding	22.58 (14)	47.37 (9)	**0.046**
Timing OGTT (months)	3.0 ± 0.57	2.7 ± 0.61	0.067
FPG (mmol/L) 30 min glucose OGTT (mmol/L) 1-h glucose OGTT (mmol/L) 2-h glucose OGTT (mmol/L)	5.3 ± 0.57 8.9 ± 1.37 9.4 ± 1.92 8.1 ± 1.32	5.4 ± 0.63 9.2 ± 1.39 9.9 ± 1.70 8.1 ± 1.36	0.222 0.374 0.136 0.769
% Metabolic syndrome	22.58 (14)	47.37 (9)	**0.046**
BMI (kg/m^2^)	27.6 ± 4.65	29.0 ± 8.34	0.612
% Overweight (BMI 25.0–29.9) % Obese (BMI ≥ 30)	36.92 (24) 32.31 (21)	5.26 (1) 47.37 (9)	**0.019**
Mean systolic blood pressure (mmHg)	118.5 ± 12.43	121.5 ± 11.78	0.146
Mean diastolic blood pressure (mmHg)	74.5 ± 10.69	77.4 ± 9.13	0.284
% Hypertension (systolic BP ≥ 140 and/or diastolic BP ≥ 90 mmHg)	7.69 (5)	21.05 (4)	0.199
% Waist circumference >80 cm	87.10 (54)	73.68 (14)	0.172
% Waist circumference > 88 cm	61.29 (38)	68.42 (13)	0.787
% PPWR > 0 kg % PPWR > 5 kg	56.92 (37) 13.85 (9)	47.37 (9) 15.79 (3)	0.601 1.000
HbA1c (%)	5.4 ± 0.36	5.5 ± 0.47	0.610
HbA1c (mmol/mol)	35.3 ± 3.93	36.1 ± 5.14	0.610
Fasting total cholesterol (mmol/L) Fasting HDL-cholesterol (mmol/L) Fasting LDL-cholesterol (mmol/L) Triglycerides (mmol/L)	4.8 ± 0.86 1.5 ± 0.38 2.9 ± 0.84 1.2 ± 0.74	5.1 ± 0.97 1.4 ± 0.36 3.2 ± 0.84 1.2 ± 0.54	0.178 0.268 0.135 0.333
Matsuda insulin sensitivity	3.8 ± 2.12	3.5 ± 2.34	0.354
HOMA-IR	2.5 ± 1.35	3.3 ± 2.86	0.413
HOMA-B	123.5 ± 72.72	130.3 ± 75.13	0.858
ISSI-2	1.4 ± 0.32	1.3 ± 0.36	0.613
Insulinogenic index/HOMA-IR	0.2 ± 0.10	0.2 ± 0.14	0.775
FFQ Total fruit (g) Total vegetables (g) Total meat (g) Total fish (g) Total discretionary foods (g) Daily protein intake (g) Daily fat intake (g) Daily carbohydrate intake (g) Daily fiber intake (g) Daily water intake (ml) Dietary Health Index (%)	130.3 ± 108.03 191.9 ± 100.64 113.5 ± 59.25 22.2 ± 19.54 319.4 ± 274.63 65.8 ± 21.27 56.8 ± 17.52 191.0 ± 61.01 18.3 ± 6.57 2122.32 ± 552.78 75.6 ± 12.07	129.8 ± 99.17 191.4 ± 97.7 111.5 ± 58.02 16.1 ± 9.11 210.6 ± 188.93 63.4 ± 18.40 53.4 ± 19.21 172.6 ± 50.51 17.2 ± 5.36 2005.2 ± 533.72 78.0 ± 10.80	1.000 0.995 0.921 0.391 0.198 0.996 0.276 0.345 0.584 0.307 0.436
IPAQ/METs category at time of OGTT % Low % Moderate % High	13.11 (8) 44.26 (27) 42.62 (26)	18.75 (3) 31.25 (5) 50.00 (8)	0.596
SF36 Physical functioning Role physical Role emotional Vitality Mental health Social functioning Pain General health	86.1 ± 20.23 77.3 ± 26.11 87.3 ± 17.19 61.4 ± 16.03 78.9 ± 10.19 89.2 ± 17.38 80.2 ± 21.99 72.5 ± 16.45	83.5 ± 21.42 67.3 ± 35.53 62.8 ± 32.96 50.4 ± 22.15 63.5 ± 18.52 67.7 ± 29.34 75.4 ± 26.28 57.7 ± 20.43	0.196 0.380 **0.004** 0.067 **0.001** **0.003** 0.579 **0.004**
STAI-6	11.2 ± 2.31	14.9 ± 4.34	**<0.001**
**One year post-randomization**			
% Breastfeeding	81.54 (53)	73.68 (14)	0.520
Timing OGTT (months)	15.1 ± 0.84	15.1 ± 0.82	0.834
FPG (mmol/L) 30 min glucose OGTT (mmol/L) 1-h glucose OGTT (mmol/L) 2-h glucose OGTT (mmol/L)	5.4 ± 0.77 8.6 ± 1.61 8.6 ± 2.50 7.3 ± 2.26	5.7 ± 0.69 9.4 ± 1.55 9.6 ± 1.53 7.8 ± 1.37	**0.033** 0.063 **0.021** 0.088
% IFG % IGT % IFG + IGT	29.41 (10) 47.06 (16) 23.53 (8)	28.57 (4) 28.57 (4) 42.86 (6)	0.411
% T2DM	6.15 (4)	5.26 (1)	1.000
% Metabolic syndrome	25.00 (16)	47.37 (9)	0.087
BMI (kg/m^2^)	27.1 ± 4.98	28.8 ± 8.96	0.688
% Overweight (BMI 25.0–29.9) % Obese (BMI ≥ 30)	35.38 (23) 27.69 (18)	0.00 (0) 47.37 (9)	**0.003**
Mean systolic blood pressure (mmHg)	117.0 ± 12.86	123.9 ± 11.83	0.054
Mean diastolic blood pressure (mmHg)	75.2 ± 9.23	80.5 ± 8.63	**0.031**
% Hypertension (systolic BP ≥ 140 and/or diastolic BP ≥ 90 mmHg)	9.23 (6)	26.32 (5)	0.114
% Waist circumference >80 cm	75.00 (48)	63.16 (12)	0.383
% Waist circumference > 88 cm	50.00 (32)	52.63 (10)	1.000
% PPWR > 0 kg % PPWR > 5 kg	52.31 (34) 16.92 (11)	36.84 (7) 5.26 (1)	0.300 0.282
HbA1c (%)	5.3 ± 0.44	5.4 ± 0.38	0.181
HbA1c (mmol/mol)	34.8 ± 4.83	35.9 ± 4.13	0.181
Fasting total cholesterol (mmol/L) Fasting HDL-cholesterol (mmol/L) Fasting LDL-cholesterol (mmol/L) Triglycerides (mmol/L)	4.9 ± 0.87 1.5 ± 0.43 2.9 ± 0.75 1.2 ± 0.87	5.3 ± 1.38 1.4 ± 0.35 3.2 ± 1.20 1.6 ± 0.71	0.317 0.231 0.351 **0.005**
Matsuda insulin sensitivity	4.0 ± 2.36	3.0 ± 2.04	0.069
HOMA-IR	2.7 ± 1.99	4.5 ± 4.94	0.196
HOMA-B	118.6 ± 53.40	138.8 ± 85.79	0.484
ISSI-2	1.7 ± 0.72	1.4 ± 0.47	0.089
Insulinogenic index/HOMA-IR	0.2 ± 0.23	0.2 ± 0.08	0.253
FFQ Total fruit (g) Total vegetables (g) Total meat (g) Total fish (g) Total discretionary foods (g) Daily protein intake (g) Daily fat intake (g) Daily carbohydrate intake (g) Daily fiber intake (g) Daily water intake (ml) Dietary Health Index (%)	137.0 ± 95.16 210.6 ± 98.91 103.9 ± 53.78 19.5 ± 20.16 313.4 ± 268.15 58.4 ± 17.76 50.0 ± 15.98 169.2 ± 56.44 16.9 ± 5.48 2044.4 ± 596.70 76.2 ± 10.97	106.6 ± 63.33 178.9 ± 113.16 99.4 ± 65.13 16.7 ± 10.92 223.5 ± 214.68 56.5 ± 16.27 46.8 ± 23.14 151.3 ± 49.85 14.9 ± 4.83 1674.1 ± 611.01 73.6 ± 10.30	0.269 0.195 0.477 0.831 0.231 0.521 0.155 0.231 0.105 **0.025** 0.199
IPAQ/METs category at time of OGTT % Low % Moderate % High	14.29 (8) 39.29 (22) 46.43 (26)	11.76 (2) 29.41 (5) 58.82 (10)	0.800
SF36 Physical functioning Role physical Role emotional Vitality Mental Health Social functioning Pain General health	90.4 ± 13.41 73.8 ± 17.72 85.4 ± 17.87 83.3 ± 12.26 73.4 ± 12.16 62.1 ± 17.95 75.7 ± 15.80 55.2 ± 14.22	80.5 ± 25.76 62.8 ± 23.98 57.0 ± 24.10 57.6 ± 16.87 50.8 ± 12.94 36.8 ± 23.38 58.4 ± 21.39 42.6 ± 10.19	0.083 **0.027** **<0.001** **<0.001** **<0.001** **<0.001** **0.002** **<0.001**
STAI-6	11.6 ± 2.66	16.4 ± 4.00	**<0.001**

GDM: gestational diabetes mellitus; PCOS: polycystic ovary syndrome; BMI: body mass index; T2DM: type 2 diabetes mellitus; OGTT: oral glucose tolerance test; BP: blood pressure; IFG: impaired fasting glucose; IGT: impaired glucose tolerance; PPWR: postpartum weight retention; HDL: high density lipoprotein cholesterol; LDL-cholesterol: low density lipoprotein cholesterol; HOMA-IR: Homeostasis Model of Assessment—Insulin Resistance; HOMA-B: Homeostasis Model of Assessment—Beta-cell Function; ISSI-2: insulin secretion-sensitivity index-2; FFQ: Food Frequency Questionnaire; IPAQ: International Physical Activity Questionnaire; METs: metabolic equivalent of task [MET] minutes/week; CES-D: Center for Epidemiologic Studies–Depression; SF-36: quality-of-life questionnaire; STAI-6: Spielberger State-Trait Anxiety Inventory. Categorical variables are presented as frequencies % (n); continuous variables are presented as mean ± SD if normally distributed and as median ± IQR if not normally distributed; Differences are considered significant at *p*-value < 0.05. Bold means a statistical significant value of *p* < 0.05.

## Data Availability

All data supporting the findings of this sub analysis of the MELINDA study are available in this article. Additional data may be provided upon reasonable request to the corresponding author. All data have been anonymized in accordance with ethical and privacy restrictions.

## Abbreviations

The following abbreviations are used in this manuscript:

GDM	Gestational Diabetes Mellitus
T2DM	Type 2 Diabetes Mellitus
MELINDA	Mobile-Based Lifestyle Intervention in Women with Glucose Intolerance after Gestational Diabetes Mellitus
RCT	Randomized Controlled Trial
IADPSG	International Association of the Diabetes and Pregnancy Study Groups
ADA	American Diabetes Association
BMI	Body Mass Index
OGTT	Oral Glucose Tolerance Test
IDF	International Diabetes Federation
IFG	Impaired Fasting Glucose
IGT	Impaired Glucose Tolerance
NAM	National Academy of Medicine
IOM	Institute of Medicine
NGT	Normal glucose tolerance
MET	Metabolic Equivalent of Task
IPAQ	International Physical Activity Questionnaire
CES-D	Center for Epidemiologic Studies Depression Scale
FFQ	Food Frequency Questionnaire
STAI-6	State-Trait Anxiety Inventory-6
RPS-DD	Risk Perception Survey for Developing Diabetes
SF-36	Quality-of-life questionnaire
HOMA-IR	Homeostasis Model of Assessment—Insulin Resistance
HOMA-B	Homeostasis Model of Assessment—Beta-cell Function
ISSI-2	Insulin secretion-sensitivity index-2
BP	Blood pressure
PPWR	Postpartum Weight Retention
HDL	High-Density Lipoprotein
LDL	Low-Density Lipoprotein
PCOS	Polycystic Ovary Syndrome
